# An Alternative to Biliverdin, Mesobiliverdin IXα and Mesobiliverdin-Enriched Microalgae: A Review on the Production and Applications of Mesobiliverdin-Related Products

**DOI:** 10.3390/molecules30061379

**Published:** 2025-03-19

**Authors:** Naveena Poudyal, Jon Y. Takemoto, Yuan-Yu Lin, Cheng-Wei T. Chang

**Affiliations:** 1 Department of Chemistry and Biochemistry, Utah State University, 0300 Old Main Hill, Logan, UT 84322-0300, USA; naveena.poudyal@usu.edu; 2 Department of Biology, Utah State University, 5305 Old Main Hill, Logan, UT 84322-5305, USA; jon.takemoto@usu.edu; 3 Department of Animal Science and Technology, National Taiwan University, Taipei City 106, Taiwan; yylin@ntu.edu.tw

**Keywords:** anti-inflammatory, antioxidative, spirulina, animal feed additive, osteoporosis

## Abstract

Despite attracting interest for decades due to its anti-inflammatory and antioxidant capabilities, the use of biliverdin IXα (BV) in medicine and agriculture is hampered by uncertain purity and limited availability. A significant amount of effort has been devoted to the production and application of BV, but with limited success. Mesobiliverdin IXα (MBV), a natural BV analog derived from microalgae, offers a path to overcome the limitations of BV. MBV production is scalable, and it can be obtained at high purity. MBV and BV share important structural features (e.g., bridging propionate groups) and both are substrates of biliverdin reductase A (BVRA), and thus exert the same mechanisms and pathways for anti-inflammatory action. To enable the use of MBV in industry, especially in agriculture, a cost-effective product, mesobiliverdin-enriched microalgae (MEM), was developed. In this review, we focus on recent developments and investigations of MBV and MEM, and compare their effectiveness with BV and Spirulina. This review article highlights cost-effective and scalable production of MEM, the therapeutic potential of MBV in cytoprotection and anti-inflammation, and MEM as an animal feed additive for improved gut health and amelioration of osteoporosis. More studies are ongoing to expand the potential applications of both MBV and MEM from fundamental research to industrial and agricultural practices.

## 1. Introduction

**Figure 1:**
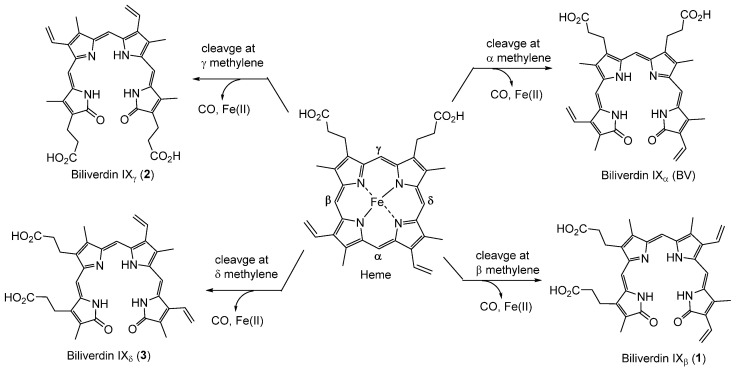
Heme, BV and biliverdin isomer produced after methene bridge cleavage by HO at the α, β, γ and δ positions. (adopted and modified from ref. [4].

Biliverdin IXα (BV) is a linear tetrapyrrole produced by ring cleavage of heme catalyzed by the enzymes, heme oxygenases (HOs) [1]. Heme degradation begins with cleavage of one of four heme methene bridges by HO, leading to the production of linear tetrapyrroles, including biliverdins and biliverdin isomers [1,2,3], CO and Fe(II) (Figure 1). The most common isomer is resulted from cleavage at the α position by canonical heme oxygenase-1 (HO-1), which occurs in animals, higher plants, algae, fungi, and bacteria to create BV [1,2]. Hence, the term “biliverdin” typically refers to the α isomer BV. Although BV is the dominant product of this enzymatic process, substrate cleavage obscurity, in part, explains the need for purifying BV, as it is the most potent anti-inflammatory isomer.

There are other sources or approaches that yield BV. For example, many non-mammalian animals accumulate heme-derived bilins that impart color, and a large fraction are biliverdins, mostly BV [3]. Engineered bacteria have been employed for the production of BV as well [5]. Nevertheless, the need for arduous purification and challenge in scale-up production remain the main obstacles preventing BV from being used clinically.

### 1.1. Cytoprotective Activities of BV

The cytoprotective capabilities of BV have attracted significant interest for decades, and diverse activities have been reported, including beneficial effects on organs (heart, kidney, liver, intestines, and lungs), organ transplantation, injury and wound healing, viral infections, Alzheimer’s disease, diabetes, osteoporosis, and intestinal bowel diseases (IBD) [6,7,8,9,10,11,12,13,14,15,16,17,18]. In red-blooded animals, BV is reduced via NADPH/NADH biliverdin reductase A (BVRA) to bilirubin IXα (BR), which, in turn is consecutively bound to serum albumin and glucuronic acid for excretion in bile or urine [5]. The overall process serves to eliminate heme—which is toxic when accumulated [19,20].

**Figure 2:**
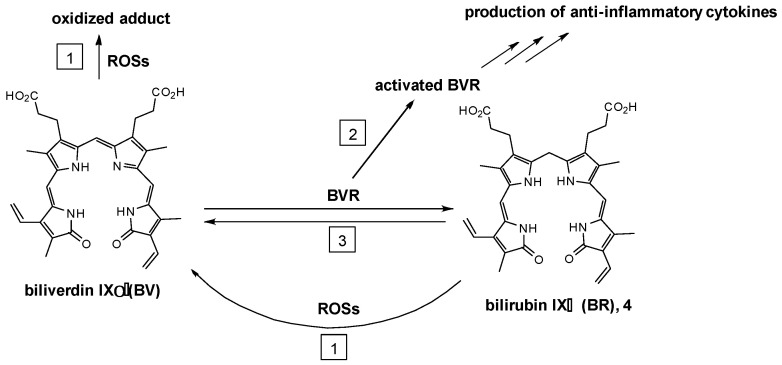
Antioxidative and anti-inflammatory MOAs of BV and BR. (1) Antioxidative effect of BV and BR; (2) activation of BVR and the expression of downstream anti-inflammatory cytokines; (3) BVR-catalyzed conversion of BV to BR.

**Figure 3:**
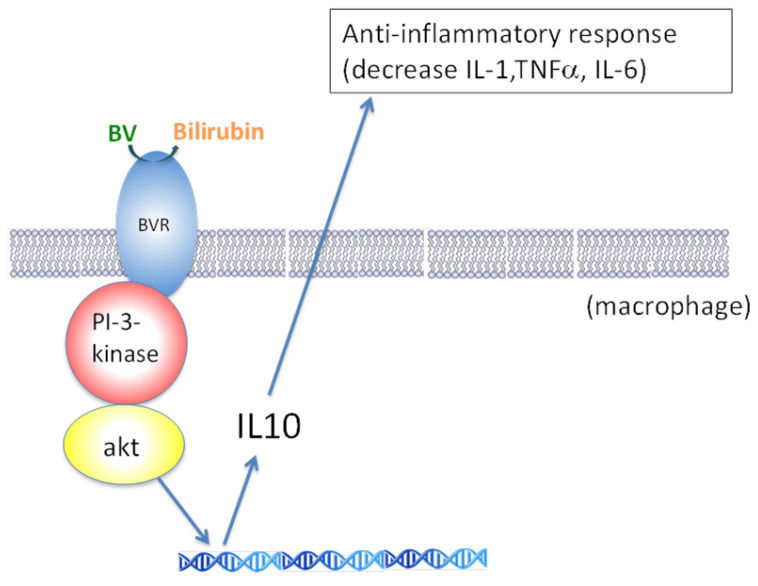
BV mediates anti-inflammatory effects via BVR signaling mechanisms. (Adopted and modified from ref. [8]).

The process of converting BV into BR via the action of BVRA is thought to contribute to the observed cytoprotective activities attributed to BV. In general, there are three modes of action (MOAs), which could work independently or in combination, (1) antioxidative effects of BV and BR; (2) activation of BVRA leading to expression of downstream anti-inflammatory cytokines; and (3) BVRA-catalyzed conversion of BV to BR (Figure 2). First, both BV and BR are strong antioxidants that can scavenge harmful reactive oxygen species (ROS), and consequently confer protection against cellular oxidative damage [19,20,21,22,23,24]. Second, BVRA is a highly multifunctional and cell regulatory enzyme [6,7]. BVRA activation via binding with BV signals downstream pathways to produce anti-inflammatory cytokines and to suppress pro-inflammatory gene expression (Figure 3) [8]. Examples of BVRA-mediated cytoprotective pathways include PI3K/Akt pathway-dependent protection against hypoxia/reoxygenation [24], regulation of anti-apoptotic transcription factor NF-κB [25], induction of anti-inflammatory cytokine interferon-10 [8], and the nitrosylation-dependent inhibition of pro-inflammatory TLR4 expression [26]. Thirdly, BR can react with ROS and in doing so is oxidized directly back to BV, which can be reduced by BVRA again. Such a redox reaction cycle suggests that BV or BR can exert their effects at catalytic or low quantities.

### 1.2. Limitations of BV as a Therapeutic Anti-Inflammatory and Antioxidant

In order for BV to become a successful anti-inflammatory and antioxidant therapy, it must be produced with high yield and at low cost and with a high degree of purity and preferably using non-mammalian resources. Currently, industrially produced BV is hampered by insufficient quantity, derivation from animal sources, and uncertain purity, mainly due to contamination from BV isomers. Despite many publications on the anti-inflammatory and therapeutic effects and production of BV, the above-mentioned problems hinder the feasibility of commercial use of BV in industries, medicine, and agriculture [5,27,28,29,30,31,32,33].

## 2. Mesobiliverdin IXα (MBV) as an Alternative to BV

### 2.1. Structural Comparison of BV, Mesobiliverdin IXα (MBV), and Phycocyanobilin (PCB)

**Figure 4:**
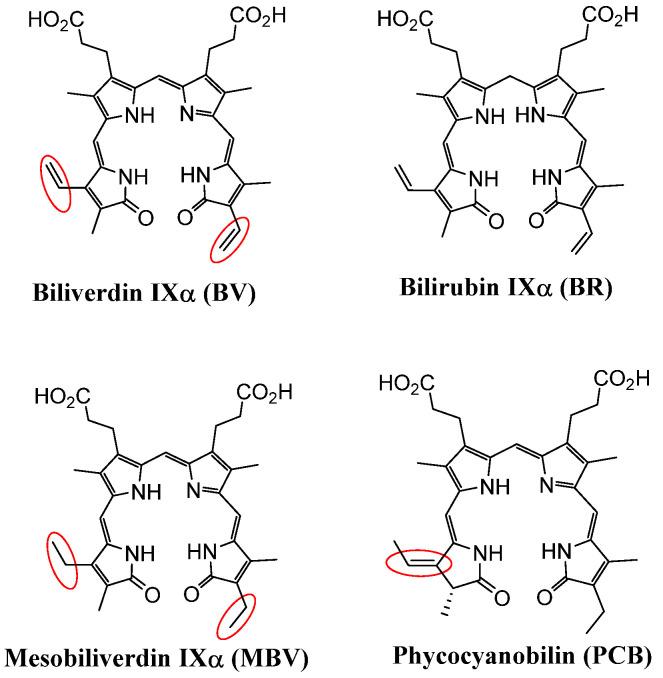
Structures of bilirubin, biliverdin, mesobiliverdin, and phycocyanobilin.

To overcome the limitations of BV as a therapeutic, mesobiliverdin IXα (MBV), a close BV analog, was purified and synthesized from microalgae phycocyanobilin (PCB) with high purity and in large amounts [4]. MBV differs from BV, as it lacks two carbon–carbon double bonds (C=C) (Figure 4). The redox potential of molecules like BV or MBV are highly dependent on the conjugation of the chemical structures (mainly double bond and lone-paired electrons). The additional carbon–carbon double bonds on BV actually belong to a “cross-conjugation” and thus do not participate in the main resonances that characterize linear tetrapyrroles. On the other hand, PCB has one carbon–carbon double bond that deviates (not conjugated) from the characteristic resonance of linear tetrapyrroles common to MBV and BV. Hence, it is expected that the redox potential and, thus, the chemical and biological properties of PCB will be drastically different from those of MBV and BV. In fact, MBV and BV are equally good substrates for human BVRA, whereas PCB does not serve as a substrate of BVRA [34]. Hence, MBV is predicted to have therapeutic potential through antioxidative and anti-inflammatory activities similar to that of BV with the added benefits of scalable production in large amounts from a non-animal source.

### 2.2. MBV Production from PCB

PCB is a major light-harvesting pigment present in the phycobiliproteins allophycocyanin and phycocyanin of microalgae, which include cyanobacteria, e.g., *Arthrospira*, *Spirulina platensis*, red algae, e.g., *Porphyridium, Gracilariopsis lemaneiformis*, glaucophytes, e.g., *Cyanophora paradoxa*, and some cryptomonads, e.g., *Hemiselmis virescens* [35,36,37]. PCB is an effective antioxidant with high oxygen radical scavenging capacity, and can regulate important markers of oxidative stress and endothelial dysfunction such as eNOS, p22NOX subunit [38,39]. Another therapeutic function of PCB includes its suppression of cancer through antiproliferative effects on cancer cells [40,41] and reduction of expression of pro-inflammatory factors like IL-6 and IFN-γ for inflammation reduction [38]. Although PCB is known for its antioxidant and therapeutic activities, the lack of detailed knowledge of its MOA, especially considering that PCB is not the substrate of BVR, imposes skepticism on the clinical applications of PCB.

Commercial Spirulina, the dried biomass of certain microalgae species, is the most economic source for producing PCB. Structurally, MBV and PCB differ in terms of the position of a carbon–carbon double-bond (Figure 4), allowing researchers at Utah State University to develop a method for the synthesis of MBV from PCB in scalable quantities and with the desired purity [4]. MBV production from Spirulina and PCB does not share the problems of low yield and contaminants that plague BV production methods.

While pure MBV is suitable and often essential for conducting small-scale research experiments such as cell-based testing, the costs of producing enough MBV to meet demands for large-scale industrial and agricultural purposes would be relatively high. Thus, a further and more cost-effective MBV product mesobiliverdin-enriched microalgae (MEM), was developed [42,43,44]. MEM contains 1–5% MBV by weight and can be produced in scalable kilogram quantities, making it suitable for applications in industry and agriculture where purer forms of MBV are not required.

## 3. Investigations and Applications of MBV and MEM

### 3.1. MBV Cytoprotection Against Oxidative Stress in Pancreatic Islet Allograft Transplantation

BV has been noted for its potential for improving pancreatic islet allograft transplantation efficacy [45,46]. Typical procedures involve excising normal islets from donor pancreata, storing the islets in preservation solutions and then injecting the islets into the intraportal ducts of type I diabetic recipients. Since such procedures are historically hindered by allograft rejection and oxidative damage of islet β-cells. Various anti-inflammatories have been examined for their abilities to enhance the rate of islet survival following islet allograft transplantation.

Both MBV and BV are equally good substrates for human BVR. MBV and BV were examined for their ability to enhance rat pancreatic islet yield for allograft transplantation into diabetic recipients (Table 1) [47]. Highlighted in Table 1, MBV increased the survival yield of islet cells 86.7% at concentrations as low as 1 μM. In contrast, BV showed a 35.5% increase at best. Higher concentrations of MBV decreased the survival yield, suggesting a possible cytotoxicity issue. In this study, MBV appears to possess therapeutic potential similar to, if not better, than that of BV.

### 3.2. MBV Amelioration of DSS-Induced Colitis

Inflammatory bowel diseases (IBDs) are enduring conditions distinguished by inflammation of the intestines and the presence of oxidative stress [48,49,50,51]. Thus, anti-inflammatory therapy has been one of the focused countermeasures against IBDs [51]. However, the majority of anti- inflammatory approaches against IBD either lack effectiveness, are expensive, or have undesirable side effects [52,53,54,55,56]. Natural anti-inflammatory compounds, such as curcumin, have been examined to relieve IBDs, but their effectiveness is often limited by poor bioavailability following ingestion [57].

Activation of BVR by BV suppresses or protects against a range of acute or chronic inflammatory disorders. For example, BV and BVR activation have been suggested as potential treatments for IBDs in human patients [53,54,55,56]. Inspired by these reports, Lin et al., investigated the therapeutic effectiveness of MBV in a mouse model of colitis that was induced by dextran sulfate sodium (DSS) [58]. Administration of DSS led to a significant decrease in body weight in mice. However, MBV reduced weight loss. Though complete restoration of body weight was not achieved, significant improvements in terms of colon weight, colon length, and the ratio of colon weight to colon length were observed.

**Figure 5:**
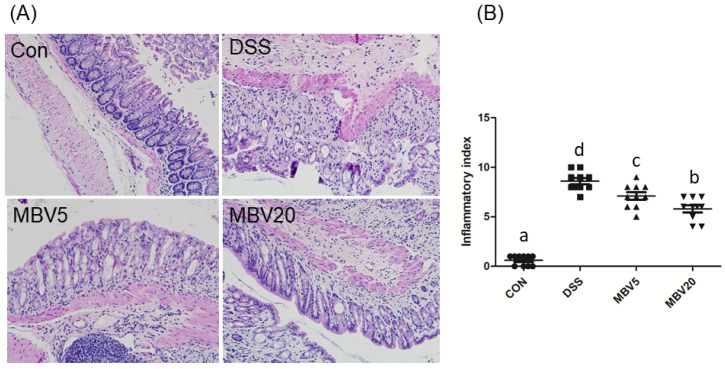
Effects of MBV on DSS-induced colitis in mice. (**A**) Histological sections of HE staining of representative colon (×100); (**B**) inflammatory index. The MBV5 group received a pretreatment with a concentration of 5 μM MBV per day per mouse for five days, while the MBV20 group received a dosage of 20 μM MBV per day per mouse. (Adopted and modified from ref. [58]).

Histological examination of colon shows that MBV administration substantially mitigated the deleterious effects induced by DSS (Figure 5A), and lowered the inflammatory index (Figure 5B). Additionally, DSS exposure elevated oxidative stress, as indicated by the increased level and activity of superoxide dismutase (SOD) and myeloperoxidase (MPO). However, diets supplemented with MBV effectively reduced the levels of SOD and MPO compared to MBV-free diets.

**Figure 6:**
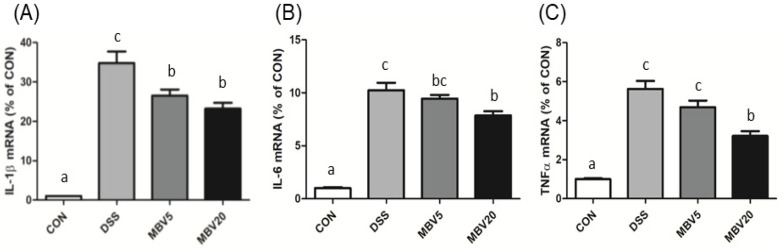
MBV treatment decreases inflammatory mediators in DSS-induced mice. The levels of (**A**) IL-1β, (**B**) IL-6, and (**C**) TNF- α- mRNA were measured by real-time PCR analyses. (Adopted and modified from ref. [58]).

Finally, real-time PCR analysis revealed that treatment with MBV led to a marked decrease in the levels of inflammatory mediators (IL-1β, IL-6 and TNF-α) compared to the DSS group (Figure 6). These findings strongly demonstrate that MBV has an anti-inflammatory role in vivo and support the use of MBV as a naturally derived therapeutic against IBD.

### 3.3. MEM Improved Chicken Gut Health and Growth Without Antibiotic Supplementation

IBDs are prevalent and cause significant economic loss and food safety concerns in agricultural livestock. For example, antibiotic-associated colitis in pigs and horses [49], Johne’s disease in cattle and other ruminants [50], and necrotic enteritis and dead gut disease in chickens [59] are diseases that have large impacts on global agro-economies.

Spirulina grown commercially in large quantities (nearly 3000 tons per year) has been used as livestock and fish feed worldwide for decades [60,61,62]. Considering the connection between PCB (isolated from Spirulina) and MBV (synthesized from PCB), it is reasonable to consider that MBV-enriched Spirulina-based feed such as MEM could be a potential remedy for IBDs. Since employing MBV directly as animal feed was not cost-effective in quantities needed by the farming industry, MEM was tested as a feed additive for farm animals. Besides its anti-inflammatory and antioxidative effects, MEM also contains nutrients from the microalgae Spirulina, including protein, carbohydrates, balanced amino acids, carotenoids, fatty acids including γ-linolenic acid, vitamins and minerals [62,63,64].

In a study reported by Chang et al., broilers fed with regular feed supplemented with MEM resulted in greater protective and beneficial effects for gut health compared to those fed with regular feed mixed with Spirulina extract or the antibiotic amoxicillin (AMX) [42]. Histological examination showed that feed supplemented with AMX decreased duodenum and ileum villi lengths below control levels while MEM increased villi lengths in the duodenum, jejunum, and ileum above AMX treatment lengths.

**Figure 7:**
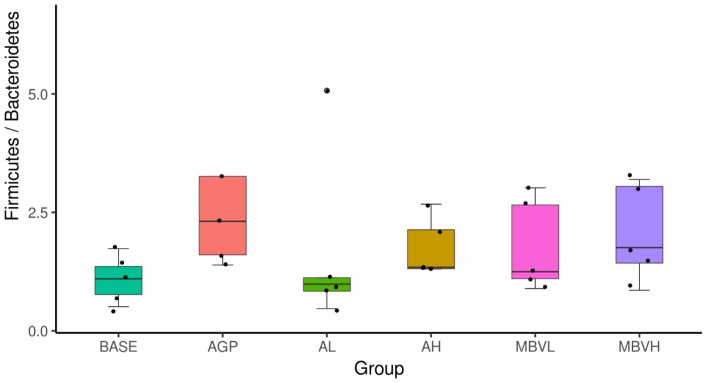
Effect of Spirulina and MEM as feed supplement on broiler gut Firmicutes/Bacteroidetes (F/B) ratios. Base: regular feed only; AGP: regular feed added with AMX (0.1% by weight), the antibiotic growth promotor; AL: regular feed added with Spirulina (0.05% by weight); AH: regular feed with added Spirulina (0.1% by weight); MBVL: regular feed with added MEM (0.05% by weight); MBVH: regular feed with added MEM (0.1% by weight). (Adopted and modified from ref. [42]).

Previous studies have shown that the gut microbiota of obese animals and humans exhibit a higher Firmicutes/Bacteroidetes (F/B) ratio compared to normal-weight individuals, thus making the F/B ratio an indicator of gaining body weight [65,66]. While high F/B ratio may not be desirable in humans due to its association with obesity, it could be beneficial for the animal farming industry. In this research using broilers, the gut microbiome F/B ratio increased in the MEM-treated group with ratios up to the level of the AMX-treated group and especially for the group that was fed with regular feed plus 0.1% of MEM (MBVH) (Figure 7). On the other hand, groups that were fed with regular feed plus Spirulina (0.05 or 0.1%) manifested F/B ratios that were much lower than the groups treated with added MEM.

Another indicator of gut health is the population of *Lactobacillus* sp. [67]. Lactobacillus species represent a major group of microbiota in humans and other animals and display mutualism with hosts by protecting them against potential invasions by pathogens. The host provides nutrients in return [68].

**Figure 8:**
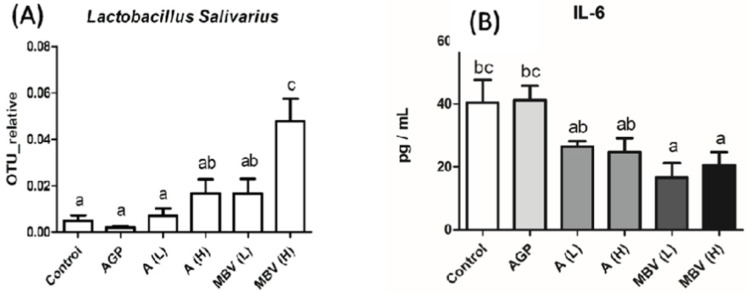
Relative abundance of *Lactobacillus salivarius* (**A**), and level of IL-6 (**B**) of broiler. OTU: operational taxonomic unit; control: regular feed only; AGP: regular feed with added AMX (0.1% by weight), the antibiotic growth promotor; AL: regular feed with added Spirulina (0.05% by weight); AH: regular feed with added Spirulina (0.1% by weight); MBVL: regular feed with added MEM (0.05% by weight); MBVH: regular feed with added MEM (0.1% by weight). (Adopted and modified from ref. [42]).

In the same study, the MEM-treated group also showed striking increases in *Lactobacillus salivarius* and decreases in the level of pro-inflammatory cytokine IL-6 in plasma (Figure 8). *L. salivarius* is a probiotic that produces bacteriocins which inhibit the growth of other bacteria and occurs in human, porcine and poultry gastrointestinal tracts [69,70,71,72]. *L. salivarius* has also been reported to modulate inflammatory cytokines against critical gut pathogens *Salmonella* and *Campylobacter jejuni* [70,71].

In these broiler experiments, MEM supplementation showed no adverse effect on growth (body weights). In short, these results demonstrate the use of cost-effective MEM as animal feed additive by promoting gut health. Furthermore, they also show that MEM is superior to Spirulina, a microalgal product that has been used in farming for decades and can eliminate the need for using antibiotics in livestock feed.

### 3.4. Gut Health Improvement of Weaning Piglets

**Figure 9:**
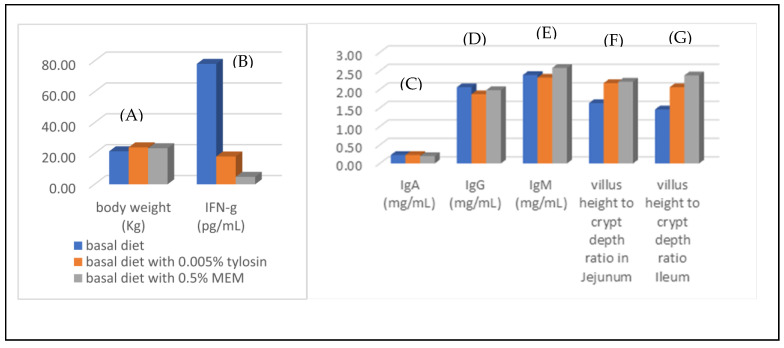
Selected data collected from the experiments of MEM in weaning piglets. (**A**) body weight of piglets; (**B**) the levels of pro-inflammatory cytokine IFN-γ in the small intestine; (**C**–**E**): immune response of piglets; (**F**,**G**): ratio of villus height to crypt depth in the jejunum and ileum (adopted and modified from ref. [44]).

Weaning is a critical period for the pig industry. The fatality of weaning animals could result from immature gastrointestinal tracts, insufficient gastric acid secretion, low intestinal enzyme activity, and poor digestibility of carbohydrates and proteins in grain-based feed in addition to environmental stress [73]. These factors can cause the propagation of intestinal pathogenic bacteria, disordering of microflora, secretion of pro-inflammatory cytokines, and diarrhea, leading to animal death [74,75,76,77]. Following the positive outcomes obtained from the broiler experiments, the same researchers investigated the use of MEM in promoting the gut health of weaning piglets [44]. The dietary treatments of weaning piglets comprised a basal diet as control, a basal diet plus 0.05% tylosin, basal diets plus 0.1% and 0.5% of MEM. All treated animals showed no adverse effects in terms of live weight, average daily gain, and feed efficiency compared to control animals (Figure 9A). Addition of MEM in basal diet reduced the levels of pro-inflammatory cytokine IFN-γ in the small intestine more effectively than the group treated with basal diet plus tylosin (Figure 9B). Stable and normal ranges of IgA, IgG, and IgM levels were observed throughout the experimental period (Figure 9C–E). Histological examination showed that adding MEM increased the ratio of villus height to crypt depth in the jejunum and ileum compared to the control and tylosin-added group (Figure 9F,G). In conclusion, feed supplementation with MEM improved gut health and lowered the secretion of inflammatory cytokines to a greater degree than that achieved with tylosin as the additive.

### 3.5. Amelioration of Osteoporosis via Promoting Osteogenic Differentiation of Mesenchymal Stem Cells

Osteoporosis is a common metabolic bone disease characterized by low bone mass, increased bone fragility, and a high risk of fracture. Current treatments for osteoporosis primarily include synthetic antiresorptive agents and bone-forming drugs. However, prolonged use of synthetic drugs can cause serious side effects, so there is an unmet need for safer and more effective treatment methods [78].

Mesenchymal stem cells (MSCs) are multipotent cells capable of differentiating into mature cells of several mesenchymal tissues, such as fat and bone [79]. Among various cell sources, bone marrow MSCs have been extensively studied in tissue regeneration and repair due to their efficient differentiation ability. In case of articular cartilage repair, human umbilical cord-derived MSCs show a greater ability to achieve proliferation and cloning [78]. A study by Vanella et al. found that the upregulation of HO-1 increases MSC-mediated osteoblasts, with a concomitant reduction in adipocytes [80]. Similarly, studies performed by other different groups on MSCs imply that employing MSCs offers a new and promising therapeutic approach to treating osteoporosis [81,82,83,84]. The role of inflammation leading to bone loss is well established [85,86,87,88]. Elevating levels of inflammation factors cause decreased differentiation of MSCs into osteoblasts (bone-forming cells), but increased differentiation to osteoclasts (bone-absorbing cells) and adipocytes [89,90,91]. Thus, MBV was examined for its effect in directing the differentiation of MSCs to osteoclasts and in bone mass restoration.

**Figure 10:**
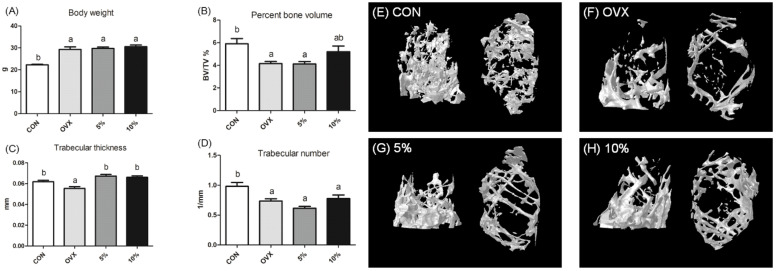
Effect of MEM on bone formation and bone mass restoration. (**A**) body weight, (**B**) bone volume, (**C**) trabecular thickness, (**D**) trabecular number, (**E**–**H**) representative metaphyseal micro-computed tomography images of mice of the indicated phenotype. CON: control; OVX: ovariectomy; 5%: 5% MEM inclusion in diet; 10%: 10% MEM inclusion in diet. (Adopted and modified from ref. [43]).

In a study by Lin et al. [43], 6-week-old female C57BL/6 N osteoporotic ovariectomized (OVX) mice were anesthetized by intraperitoneal treatment of tribromoethanol (240 mg/kg). The mice were fed with MEM-inclusion diets (5 and 10%). The results showed that no significant changes were observed in body weight across the OVX group or 5–10% MEM-treated groups, indicating no adverse effect on animal health (Figure 10A). The group fed with 10% MEM demonstrated a significant improvement in overall bone proportion compared to the OVX group with increased bone volume and trabecular thickness and number (Figure 10B–D). These results were confirmed by the representative images from the micro-CT analysis (Figure 10E–H).

**Figure 11:**
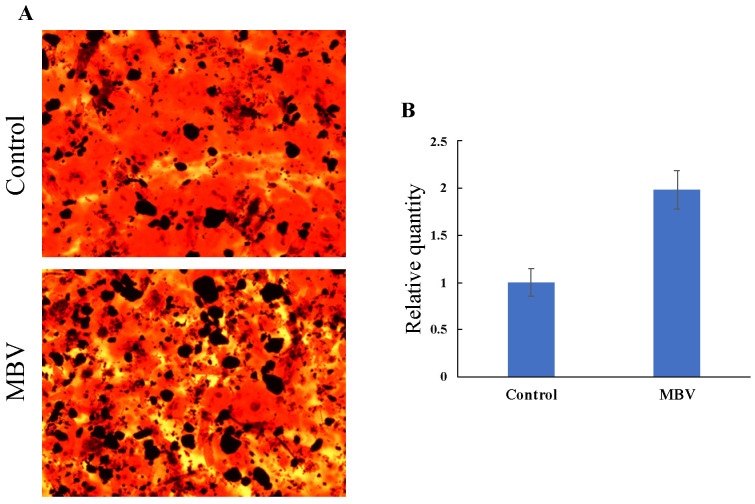
Effect of MBV on osteogenic differentiation of MSCs. (**A**) At 21 days of differentiation, the cultures showed the presence of mineralized nodules following Alizarin Red staining analysis; (**B**) relative quantified stained areas of osteocytes. Control: growth medium without MBV; MBV: growth medium with 5 μM MBV. (Adopted and modified from ref. [43]).

In support of the observed MBV-induced osteogenic differentiation of MSCs, the MSCs were grown in medium supplemented with 5 μM MBV for 3 weeks. Osteogenic differentiation of MSCs was noted in medium with and without the addition of MBV (Figure 11). However, the MBV-treated group had a two-fold increase in osteogenic differentiation of MSCs compared to the non-MBV-treated control group (Figure 11B). The augmenting effect of MBV on MSC differentiation into the osteogenic lineage was clearly demonstrated.

## 4. Conclusions and Future Research Perspectives

Spirulina has been used as animal feed in farming and aquaculture for decades, but the two main challenges are as follows: (1) relatively high production costs and (2) inconclusive effectiveness in promoting animal growth and health [92,93]. Spirulina remains relatively expensive to produce compared to other protein feed, such as soybean meal. This cost-of-production challenge is exacerbated by fully replacing the regular diet or meal with Spirulina, or mixing it with regular diet or meal in high percentages (10% or higher). For the second challenge, numerous reports show inconsistent or contradicting outcomes when Spirulina is used as feed [94,95,96,97,98,99,100]. One of the reasons could be that there are no clear molecular or mechanism-based MOA findings that identify the specific ingredients or compounds that explain the beneficial effects of Spirulina.

Spirulina may contain up to 60–70% protein, of which phycocyanin constitutes approximately 20–25% of the total biomass [101,102]. Since PCB is the main light-harvesting pigment found in the phycobiliproteins allophycocyanin and phycocyanin, numerous studies have focused on the biological activities and redox properties of PCB. Nevertheless, these studies often report only on “test-and-observe” results while providing no information as to how PCB acts as an anti-inflammatory or antioxidative molecule, and what enzyme(s) or pathway could be involved in the observed results.

In contrast, BV, which is structurally similar to PCB, has been shown to have profound anti-inflammatory or antioxidative effects that correlate with the associated health benefits. Furthermore, the MOA of BV in combination with the activity of BVR is well established. Considering that PCB is not a good substrate for BVR, it is not surprising that employing Spirulina and PCB in animal feed yields inconsistent results. The main drawback of using BV in agriculture and medicine is the cost of production pertaining to the aspects of purity and scale.

The above shortcomings of Spirulina, PCB, and BV reveal the superiority of MBV as a beneficial and therapeutic linear tetrapyrrole for applications in medicine and agriculture. Firstly, MBV shares the same MOAs as BV, as it is the substrate of BVR, while PCB, the main light-harvesting pigment, is not. Secondly, MBV production from PCB can be scaled up as a single and active isoform, unlike BV, which is hard to produce at industrial scales. Thirdly, MBV and MEM can be applied in low doses (0.05–5%) that still retain prominent anti-inflammatory effects, promoting animal health and growth. In contrast, most of the reported studies using Spirulina in feed require relatively large amounts to achieve beneficial growth and health. Finally, to the best of our knowledge, there is no BV-containing or related product that has been reported. On the other hand, MEM represents a cost-effective MBV-containing product suitable for implementation in the market.

Previous studies have indicated that incorporating MBV or MEM into feed may play a significant role in regulating physiological functions by influencing the gut microbiota. Furthermore, MBV and MEM have demonstrated their potential as practical products to meet demands for diverse therapeutic and agricultural uses. While MBV is suitable for small-scale applications such as cellular-level research investigations of mechanisms and medical therapeutics, MEM is more applicable for large-scale agricultural and related industrial applications. Future research should prioritize understanding the mechanisms by which MBV modulates microbiota composition and the subsequent physiological benefits it provides. In summary, MBV and MEM together are products for a wide scope of applications from fundamental research to industrial and agricultural practices.

## Figures and Tables

**Table 1:** Islet cell yields from pancreata infused with BV ^a^ and MBV (adopted and modified from ref. [47]).

Compound	Concentration (mM)	# Islet Cells (IEQ)	% Increase over Control
BV	1	1345 ± 629	4.3
	10	1603 ± 1073	24.4
	100	1759 ± 703	35.5
	control	1289 ± 559	
MBV	1	1599 ± 475	86.7
	10	1318 ± 805	54.0
	100	1535 ± 287	79.3
	control	856 ± 229	

^a^ Biliverdin IX_α_-HCl purchased from Frontier Specialty Chemicals, Inc., Logan, UT, USA.
